# LS Channel Estimation and Signal Separation for UHF RFID Tag Collision Recovery on the Physical Layer

**DOI:** 10.3390/s16040442

**Published:** 2016-03-26

**Authors:** Hanjun Duan, Haifeng Wu, Yu Zeng, Yuebin Chen

**Affiliations:** School of Electrical and Information Technology, Yunnan University of Nationalities, 650500 Kunming, China; hansonduan@foxmail.com (H.D.); yv.zeng@gmail.com (Y.Z.); cybuestc@sina.com (Y.C.)

**Keywords:** RFID, tag collision, channel estimation, signal separation, least-square

## Abstract

In a passive ultra-high frequency (UHF) radio-frequency identification (RFID) system, tag collision is generally resolved on a medium access control (MAC) layer. However, some of collided tag signals could be recovered on a physical (PHY) layer and, thus, enhance the identification efficiency of the RFID system. For the recovery on the PHY layer, channel estimation is a critical issue. Good channel estimation will help to recover the collided signals. Existing channel estimates work well for two collided tags. When the number of collided tags is beyond two, however, the existing estimates have more estimation errors. In this paper, we propose a novel channel estimate for the UHF RFID system. It adopts an orthogonal matrix based on the information of preambles which is known for a reader and applies a minimum-mean-square-error (MMSE) criterion to estimate channels. From the estimated channel, we could accurately separate the collided signals and recover them. By means of numerical results, we show that the proposed estimate has lower estimation errors and higher separation efficiency than the existing estimates.

## 1. Introduction

Ultra-high frequency (UHF) radio frequency identification (RFID) is a non-contact electronic identification technology [1]. UHF RFID has a lot of advantages, such as long communication range, high security, large storage capacity, and so on. Additionally, it is easily integrated into enterprise management information systems. As UHF RFID is widely used in various kinds of information systems, it becomes one of key technologies to identify objects in the Internet of Things. In a passive UHF RFID system, an RFID reader identifies multiple tags on a shared wireless channel. When the multiple tags simultaneously transmit their signals to the reader, collisions will happen [2]. Many conventional anti-collision algorithms resolve the problem only on a media access control (MAC) layer [3,4,5,6,7,8,9]. The algorithms consider the collided signals as useless information, so their identification efficiency is not high.

In recent years, an MAC-physical (MAC-PHY) cross-layer approach [10,11,12,13,14,15] is introduced. The approach combines random multiple access on an MAC layer with signal separation on a PHY layer to resolve the tag collisions. The idea uses the random multiple access to prevent tag collision on the MAC layer. If there are still some collided tags, they will then be separated on the PHY layer. In the approach, the collided signals are not longer considered as useless information. Thus, the approach has higher communication efficiency than pure MAC layer methods. For the cross-layer approach, the estimation of the wireless channel coefficient is an important issue. Good channel estimation will help to correctly recover the collided tag signal on the PHY layer. However, the channel estimation in a UHF RFID system is some different from that in other wireless communication systems. First, the estimation has to be performed under unsynchronized condition. Each UHF RFID tag has different symbol period and delay [16]. The passive tag can not synchronize its backscattering symbols due to its simple circuit [17]. Second, pilot-based channel estimation can not be performed in the RFID system. Sometimes, there are no pilots at all, e.g. TRext = 0 in EPC C1 Gen2 [16]. Moreover, we cannot alter the pilots to adapt the channel estimation because they are pre-designed.

Constellation mapping (CM) [10] is an algorithm proposed to recover the collided tag signals on the PHY layer. The algorithm maps collided signals to an orthogonal/phase (IQ) plane and then recovers the mapped signals through an unsupervised clustering method. Since there is no channel estimation in the algorithm, it is actually a blind method. Its computational complexity increases with the number of the collided tags. When the number of the collided tags is beyond two, especially, the algorithm is very difficult to separate the collided signals. The single-antenna zero-forcing (SAZF) algorithm [11] can also recover the collided signals on the PHY layer. The algorithm is not a blind method and, thus, has lower computational complexity since it uses the channel information. SAZF projects collided signals onto an orthogonal space of the signals and then searches an extreme value to estimate the channel. Under a single-receiving-antenna environment, however, the algorithm can estimate the channel for only two collided tags. When the number of tags is beyond two, SAZF does not give a solution. Successive-interference-cancel (SIC) algorithm [12] can recover more than two collided tag signals on the physical layer. In the algorithm, each step of the interference cancelation requires accurate channel information. However, the SIC algorithm’s channel estimate (SCE) adopts an inner-product method, which will produce accumulated errors. The errors will degrade the performance of the estimate when the number of collided tags increases. The least-squares channel estimate based on preambles (LCE) algorithm [13] uses the method of the inner product to estimate channels. The algorithm can estimate the channels of more than two collided tags. Unfortunately, the estimated precision of the algorithm is not high.

In this paper, we propose a channel estimation algorithm called orthogonal-matrix least-square channel estimate (OLCE). Since the preambles are known for a reader, the reader can use the information of preambles to obtain an orthogonal matrix under minimum mean square errors (MMSE) criterion. The algorithm can accurately estimate the channel coefficients of more than two collided tags. From the estimated channel coefficients, then, we recover the UHF RFID tag collision on the PHY layer. Through numerical results, the estimation errors of the algorithm are lower than the existing algorithms, and the separation efficiencies of the proposed algorithm are higher than the existing algorithms.

## 2. Algorithm Section

### 2.1. System Model

In this paper, we consider a basic communication between several tags and an RFID reader equipped with a single receiving antenna. During the communication, the reader does not modulate any signals. It provides the RFID tags with energy in the form of a continuous carrier transmission. For transmitting signals to the reader, tags use backscatter modulation. Given *N* tags transmitting in a certain slot, each tag *n*, *n* = 0,1,…*N* − 1 changes from absorbing energy to reflecting energy, by mismatching their antenna input impedance. After receiving the *N* tag collided signal, the reader downconverts the receive signals to the baseband. Hence, the complex-valued baseband signal at the receive antenna is [11,12]:

(1) $$z_{L}(t)=\sum_{n=0}^{N-1}h_{n}c_{n}(t)+L+\xi (t)$$

where $h_{n}=h_{n}^{f}h_{n}^{b}\sqrt{\Delta \sigma_{n}}$ is a flat fading linear time invariant channel in a very short time communication [12] in which $h_{n}^{f}$ denotes a forward channel (the reader to the tag *n*) and $h_{n}^{b}$ a backward channel (the tag *n* to the reader) coefficient, Δ*σ_n_* is normalized differential radar cross section; *ξ*(*t*) is additive white Gaussian noise added at the reader; $c_{n}(t)=\sum_{k=0}^{K-1}d_{n,k}g(t-ka_{n}-b_{n})$ realizes an on-off key, and features different symbol period *a_n_* and symbol delay *b_n_* [14,18], *K* is the length of symbol block, *d_n,k_* ∈ {0,1} denotes the transmitted symbol and *g*(*t*) denotes the pulse modulation signal.

In EPC C1 Gen2 [16] and ISO18000-6 [19] standards, there is a quiet period before tags reflect the signal. In this period, all tags absorb energy. The reader only discovers the carrier leakage *L*, *i.e.*, *Z_L_*(*t*) ≈ *L* when *d_n,k_* = 0. Such a period is also defined in [11] before the tags respond. We can utilize this period to estimate the carrier leakage. Hence, we make *Z*(*t*) = *Z_L_*(*t*) − *L*, the Equation (1) can be expressed as:

(2) $$z(t)=\sum_{n=0}^{N-1}h_{n}c_{n}(t)+\xi (t)$$

### 2.2. The Related Work of Channel Estimation

The problem of channel estimation in UHF RFID systems actually is how to estimate the channel coefficient *h_n_* from the collided signals *Z*(*t*).

**Figure 1:**
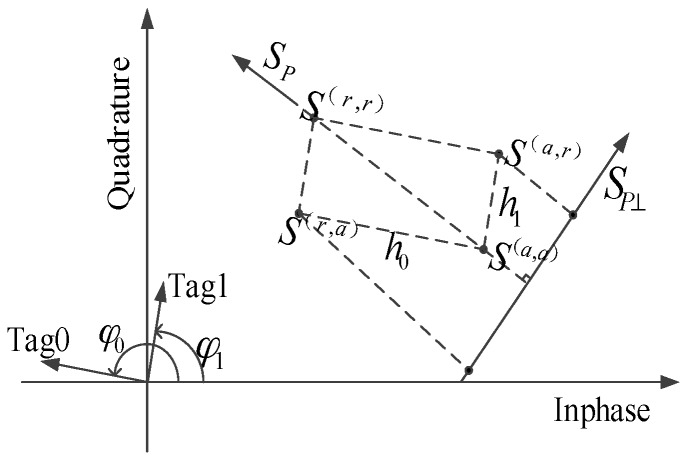
Mapping two collided tag signals to an IQ plane under a single receiving antenna.

The SAZF algorithm maps the collided signals to an inphase/quadrature (IQ) plane. The IQ plane for two collided signals is shown in Figure 1. According to [11], the channel coefficients of two tags can be estimated by:

(3) $$\begin{array}{l} h_{0}=S^{\left(r,a\right)}-S^{\left(a,a\right)}=\underset{k}{\text{min}}\{S_{P⊥}[k]\}-S^{\left(a,a\right)}\\ h_{1}=S^{\left(a,r\right)}-S^{\left(a,a\right)}=\underset{k}{\text{max}}\{S_{P⊥}[k]\}-S^{\left(a,a\right)} \end{array}$$

where

*S*^(*a*,*a*)^ = *L* denotes a state which both tags absorb;
*S*^(*r*,*a*)^ = *L* + *h*_0_ and *S*^(*a*,*r*)^ = *L* + *h*_1_ denote states which one absorbs and the other reflects;
*S*^(*r*,*r*)^ = *L* + *h*_0_ + *h*_1_ denotes a state which both tags reflect; and
$S_{P_{⊥}}$ is the orthogonal subspace of a signal space *S_P_* shown in Figure 1.

From Equation (3), SAZF can estimate the channel coefficients for two collided tags. However, when the number of tags is beyond two, SAZF cannot estimate the channel coefficients. Since the estimated equation is indeterminate, the multi-antenna technology can solve the problem [11,20]; however, this would increase the size of reader and cost.

SCE adopts SIC technique to estimate the channel coefficients. In SCE, the *n*-th tag’s channel coefficient can be estimated by [12]:

(4) $${\hat{h}}_{n}=\langle z_{n}(t),\phi_{a_{n},b_{n}}(t)\rangle /\langle \phi_{a_{n},b_{n}}(t),\phi_{a_{n},b_{n}}(t)\rangle$$

(5) $$z_{n}(t)=z_{n-1}(t)-h_{n-1}\phi_{a_{n-1},b_{n-1}}(t)$$

where *h*_0_ > *h*_1_ > … *h*_*N*−1_, *n* = 0,1,…,*N* − 1 and initial *z*_0_(*t*) = *Z*(*t*); 〈•〉 denotes the inner product computation; *φ*(*t*) ∈ {0,1} is a mother function and has the same structure as preamble signals which are known for a reader. $\phi_{a_{n},b_{n}}(t)=\phi [\left(t-b_{n}\right)/a_{n}]$ denotes a daughter function of the mother function *φ*(*t*).

The parameters *a_n_* and *b_n_* in Equation (4) are unknown and need to be estimated. Generally, the tag modulation frequency will drift over time. However, the duration of the preamble is very short and *a_n_* could be consider invariant within the duration [15]. Then, *a_n_* and *b_n_* can be estimated by [12]:

(6) $$(a_{n},b_{n})=\text{arg}\underset{\alpha \in A,\beta \in B}{\text{max}}{\langle z_{n}(t),\phi_{\alpha ,\beta }(t)\rangle }^{2}$$

where *A* and *B* denote the search ranges of *α* and *β*, respectively. Note that the estimated result in Equation (6) should be the symbol period and delay of the tag with the strongest preambles in *z_n_*(*t*). The algorithm can estimate the channel coefficients base on the signal strength of the collided tags, cumulative errors increase when the number of collided tags increases. Therefore, the estimated performance would degrade when the number of tags increases.

LCE uses the information of the preambles and an LS criterion to estimate the channel coefficients. Since the preambles are known, we can create a daughter function $\phi_{u_{m},v_{m}}\left(t\right)$ which has the same structure as the preamble $\phi_{a_{n},b_{n}}\left(t\right)$, where *u_m_* and *v_m_* are random numbers choose from the ranges *A* and *B*, respectively, and let *y_m_* denote the inner product of *Z*(*t*) and $\phi_{u_{m},v_{m}}(t)$, *i.e.*:

(7) $$y_{m}=\int_{-\infty }^{\infty }Z(t)\phi_{u_{m},v_{m}}(t)dt$$

From Equations (2) and (7), we will have:

(8) $$y_{m}=\sum_{n=0}^{N-1}h_{n}\int_{-\infty }^{\infty }\phi_{a_{n},b_{n}}\left(t\right)\phi_{u_{m},v_{m}}\left(t\right)dt+\int_{-\infty }^{\infty }\xi \left(t\right)\phi_{u_{m},v_{m}}\left(t\right)dt$$

If:

(9) $$p_{n,m}=\int_{-\infty }^{\infty }\phi_{a_{n},b_{n}}\left(t\right)\phi_{u_{m},v_{m}}\left(t\right)dt$$

(10) $$\xi_{m}=\int_{-\infty }^{\infty }\xi \left(t\right)\phi_{u_{m},v_{m}}\left(t\right)dt$$

We can change Equation (7) into:

(11) $$y_{m}=\sum_{n=0}^{N-1}h_{n}p_{n,m}+\xi_{m}$$

Hence, Equation (11) could be written in a matrix form as:

(12) Y=PH+Ξ

Where:

(13) $$Y={\left[y_{0},y_{1},\ldots y_{M-1}\right]}^{T}$$

(14) $$H={\left[h_{0},h_{1},\ldots h_{N-1}\right]}^{T}$$

(15) $$\Xi ={\left[\xi_{0},\xi_{1},\ldots \xi_{M-1}\right]}^{T}$$

(16) $$P=\left[\begin{array}{cccc} p_{0,0} & p_{0,1} & \cdots & p_{0,N-1}\\ p_{1,0} & p_{1,1} & \cdots & p_{1,N-1}\\ \vdots & \vdots & \ddots & \vdots \\ p_{M-1,0} & p_{M-1,1} & \cdots & p_{M-1,N-1} \end{array}\right]$$

Thus, the channel coefficient vector can be given by LS estimation, then [13]:

(17) $$\hat{H}=P^{+}Y$$

where **P**^+^ denotes the pseudo inverse of **P**. Here, the matrix **P** in Equation (16) should be column full rank. Thus, we should guarantee *M* ≥ *N*. Although LCE could estimate the channels of more than two collided tags, the estimation errors would not be lower from the numerical results in [13].

### 2.3. OLCE Algorithm

In this subsection, we will describe our OLCE algorithm, which could estimate the channels of more than two tags. What’s more, the algorithm has the minimum MSE. OLCE uses the information of the preambles. From Equations (1) and (2), the received signal within the duration of the tags’ preambles could be written as:

(18) $$z(t)=\sum_{n=0}^{N-1}\phi_{a_{n},b_{n}}(t)h_{n}+\xi (t)$$

where *t* ∈ [0,*T*] and *T* is one minimum preamble duration in all collided tag. We change Equation (18) into a matrix form as:

(19) Z=XH+Ξ

where:

$Z={\left[z(t_{0}),z(t_{1}),\ldots ,z(t_{M-1})\right]}^{T}$;
$X=[x_{0},x_{1},\ldots ,x_{N-1}]$;
$x_{n}={\left[x_{n}(t_{0}),x_{n}(t_{1}),\ldots ,x_{n}(t_{M-1})\right]}^{T}$, *n* = 0,1,…,*N* − 1
$x_{n}(t_{m})=\phi_{a_{n},b_{n}}(t_{m})$, m=0,1,…,M−1
$H={\left[h_{0},h_{1},‥,h_{N-1}\right]}^{T}$
$\Xi ={\left[\xi (t_{0}),\xi (t_{1}),\ldots ,\xi (t_{M-1})\right]}^{T}$

If **X** is a matrix of full column rank, we have the estimated channel $\hat{H}=X^{+}Z$ by LS, where **X**^+^ denote the pseudo-inverse of **X**. Hence, the MSE of the estimation could be derived as [21]:

(20) $$\text{MSE}=\frac{E\{{\left\|\hat{H}-H\right\|}^{2}\}}{N}=\frac{\text{tr}[X^{+}E(\Xi \Xi^{H}){X^{+}}^{H}]}{N}=\frac{\sigma^{2}}{N}\text{tr}\{{\left(X^{H}X\right)}^{-1}\}$$

where $E(\Xi \Xi^{H})=\sigma^{2}I_{M\times M}$ and *σ*^2^ is the variance of the white noise. In order to obtain the minimum MSE in Equation (20), we require $X^{H}X=\sigma_{1}^{2}I_{N\times N}$ where $\sigma_{1}^{2}$ is a constant [21]. In this scenario, the matrix **X** is orthogonal. Next, we require obtaining the matrix.

First, we show a composite signal as:

(21) $$y(t)=\sum_{n=0}^{N-1}\gamma_{n}\phi_{a_{n},b_{n}}(t)$$

where *γ_n_*, n=0,1,…N−1 is defined as matched coefficients. The coefficients could be selected as any numbers as long as $\gamma_{n_{1}}\neq \gamma_{n_{2}}\neq \gamma_{n_{3}}+\gamma_{n_{4}}\neq \gamma_{n_{5}}+\gamma_{n_{6}}+\gamma_{n_{7}}\neq \ldots$ when $n_{1}\neq n_{2}\neq n_{3}\ldots$ and so on, where *n_j_* ∈ {0,1,…*N* − 1}. From Equations (18) and (21), the composite signal *y*(*t*) is the same as *z*(*t*), except *γ_n_* ≠ *h_n_*. Since *a_n_* and *b_n_* could be estimated from Equation (6), the value of *y*(*t*) would be known. Then, we detect the time $t_{j}^{n}$ when the value of the composite signal is equal to *γ_n_*. That is $y(t_{j}^{n})=\gamma_{n}$, *j* = 0,1, …*J* − 1 where *J* is the number of times when the value is *γ_n_*. Thus, Equation (19) could be changed into:

(22) $$\overset{⌢}{Z}=\overset{⌢}{X}H+\overset{⌢}{\Xi }$$

where:

$\overset{⌢}{X}=[{\overset{⌢}{x}}_{0},{\overset{⌢}{x}}_{1},\ldots ,{\overset{⌢}{x}}_{N-1}]$
${\overset{⌢}{x}}_{n}=[x_{n}(t_{0}^{0}),x_{n}(t_{1}^{0}),\ldots x_{n}(t_{J-1}^{0}),x_{n}(t_{0}^{1}),x_{n}(t_{1}^{1}),\ldots x_{n}(t_{J-1}^{1})\ldots x_{n}(t_{0}^{N-1}),x_{n}(t_{1}^{N-1}),\ldots x_{n}(t_{J-1}^{N-1}){]}^{T}$, *n* = 0,1,…,*N* − 1
$x_{n}(t_{j}^{n})=\varphi_{a_{n},b_{n}}(t_{j}^{n})$, *j* = 0,1,…,*J* − 1

Correspondingly, $\overset{⌢}{Z}$ and $\overset{⌢}{\Xi }$ is constituted by $z(t_{j}^{n})$ and $\xi (t_{j}^{n})$, respectively. Since $y(t_{j}^{n})=\gamma_{n}$, only one item of $\varphi_{a_{n},b_{n}}(t_{j}^{n})$, *n* = 0, 1, …, *N* − 1 is 1 and the others are all 0. That is, only one column is 1 and the others are all 0, for the (*n* + 1)(*j* + 1)-th row in the matrix $\overset{⌢}{X}$. For example, if *N* = 2 and *J* = 3, then $\overset{⌢}{X}$ = [1 0;1 0; 1 0; 0 1; 0 1; 0 1]. Hence, we have $X^{H}X=JI_{N\times N}$. The minimum MSE in Equation (20) could be obtained. Therefore, the estimated channel by LS can be given by:

(23) $$\begin{array}{c} \overset{⌢}{Z}=\overset{⌢}{X}H+\overset{⌢}{\Xi }\\ \hat{H}={\overset{⌢}{X}}^{+}\overset{⌢}{Z} \end{array}$$

### 2.4. The Performance Analysis of Channel Estimation

In this subsection, we will analyze the performance of the above channel estimation.

**Figure 2:**
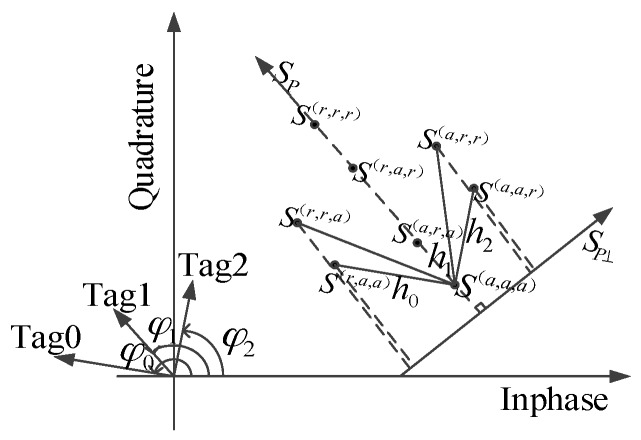
Mapping three collided tag signals to an IQ plane under a single receiving antenna.

SAZF can estimate the channel coefficients for two collided tag. However, when the number of tags is beyond two, SAZF can not estimate the channel coefficients. The I/Q plane for three collided signals is shown in Figure 2. From Equation (3), $\underset{k}{\text{min}}\left\{S_{⊥}\left[k\right]\right\}-S^{\left(a,a,a\right)}=S^{\left(r,r,a\right)}-S^{\left(a,a,a\right)}=h_{0}+h_{1}$, where $S^{\left(a,a,a\right)}$ denotes a state which three tags absorb and $S^{\left(r,r,a\right)}$ denotes a state which two tags reflect and anther one absorbs. The result of the equation above is the superposition of two channel coefficients. Thus, the equation is indeterminate.

SCE adopts an inner-product method, which will produce accumulated errors. The more the number of collided tags, the greater the accumulated errors. Specific analysis as follows, substituting *n* = 0 into Equation (4), we have:

(24) $${\hat{h}}_{0}=h_{0}+\langle \sum_{n=1}^{N-1}h_{n}\phi_{a_{n},b_{n}}(t)+\xi (t),\phi_{a_{0},b_{0}}(t)\rangle /\langle \phi_{a_{0},b_{0}}(t),\phi_{a_{0},b_{0}}(t)\rangle$$

It is seen that the second item in the right side of Equation (24) is estimated error, which will be accumulated onto ${\hat{h}}_{1},{\hat{h}}_{2},‥{\hat{h}}_{N-1}$. Therefore, the estimated performance would degrade when the number of tags increases.

LCE adopt the method of inner product to structure observation matrix . This greatly reduces the computational complexity when using the LS to solve the pseudo-inverse matrix **P**^+^. However, the estimation precision of LCE algorithm is far lower than OLCE algorithm. This is because *M* of the observation matrix is lesser, and does not meet $P^{H}P=\sigma_{2}^{2}I_{N\times N}$ where $\sigma_{2}^{2}$ is a constant. This makes the channel estimation of LCE algorithm not have the minimum MSE.

However, the OLCE algorithm uses the minimum MSE criterion, its observation matrix $\overset{⌢}{X}$ meets ${\overset{⌢}{X}}^{H}\overset{⌢}{X}=JI_{N\times N}$. This results in a smaller estimation error. What is more, due to the construction of orthogonal matrix, it does not need to make use of all of the information of the preamble, greatly reducing the computational complexity. The OLCE and LCE algorithms are using the LS criterion to estimate channel coefficients, but OLCE’s estimation error is far lower than LCE’s.

### 2.5. Signal Separation

Next, we would recover collided tag signals through the channel coefficients. Firstly, we project the collided signals to an IQ plane and get a constellation. Then, we could find several clustering centers which could be obtained from the channel coefficients. When the number of the collided tags is three, e.g., the received signal model becomes:

(25) $$Z(t)=h_{0}c_{0}(t)+h_{1}c_{1}(t)+h_{2}c_{2}(t)+\xi (t)$$

**Figure 3:**
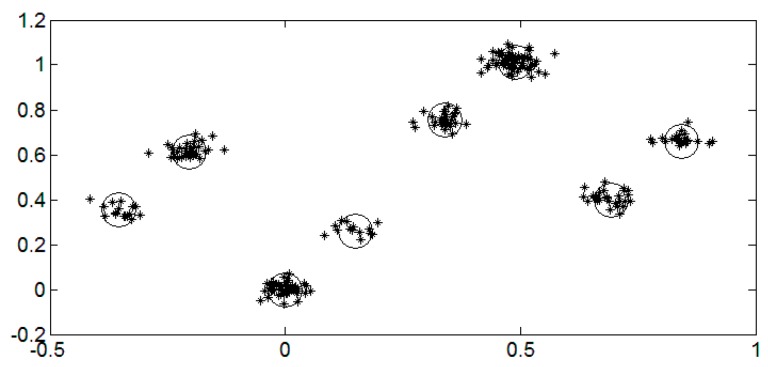
A constellation for three collided tags in an IQ plane under SNR = 20 dB.

In this case, there are eight clustering centers, $h_{0}+h_{1}+h_{2},h_{0}+h_{1},h_{0}+h_{2},h_{1}+h_{2},h_{0},h_{1},h_{2}$, and 0. Through calculating Euclidean distances between each sample point of *Z*(*t*) and the clustering centers, respectively, we would make a decision for each of tag signals. The example of three collided tags is shown in Figure 3.

In summary, we give the steps of the algorithm and a part of pseudo-code as follows:

1. Estimate symbol period *a_n_* and symbol delay *b_n_* from Equation (6);

2. Obtain the signal *y*(*t*) in Equation (21) and detect the time $t_{j}^{n}$ when $y(t_{j}^{n})=\gamma_{n}$, j=0,1,…J−1;

Set *γ*_0_ = 1, *γ*_1_ = 2, *γ*_2_ = 4, …, *γ_n_* = 2^*n*−1^
Obtain the signal *y*(*t*)
for *t* = 1:T
 if *y*(*t*) == *γ*_0_
  *j* = m_0_;
  $t_{j}^{n}=t$;
  m_0_++;
 end
 if *y*(*t*) == *γ*_1_
  *j* = m_1_;
  $t_{j}^{n}=t$;
  m_1_++;
 end
 …
 if *y*(*t*) == *γ_n_*
  *j* = m_n_;
  $t_{j}^{n}=t$;
  m_n_ ++;
 end
end

3. According to the time $t_{j}^{n}$, obtain the orthogonal matrix $\overset{⌢}{X}$ and the observation Equation (22);

for *m* = 1:N
 for *n* = 1:N
  for *j* = 1:J
  p = (*n* − 1) × J + *j*;
  X(p,m)= $\phi_{a_{m-1},b_{m-1}}(t_{j}^{n})$;
  end
 end
end

4. Estimate the channel $\hat{H}$ from Equation (23);

H_est = pinv(X) × $z(t_{j}^{n})$

5. Compute several clustering centers in an IQ plane from the estimated channel;

For example, The number of collided tags is two,

clustering centers = [ H_est(1) + H_est(2); H_est(1); H_est(2); 0];

6. Through calculating Euclidean distance between each sample point of the collided signals and the clustering centers, separate the collided signals.

for q = 1: length(clustering centers)
 distance = Euclidean distance (*z*(*t*), clustering centers(q));
end
[v,c] = min(distance);
separate the collided signals

## 3. Results and Discussion

### 3.1. System Settings

We evaluate the performance of the proposed algorithm by numerical experiments. In the experiments, we consider a scenario with a single-receive-antenna reader and some passive tags. The number of tags is from 10 to 600. When multiple tags select a time slot simultaneously, the tag signals will collide with each other. Then, we will estimate the channel coefficients and separate the collided tags. We individually perform each experiment 5000 times, and average 5000 experiment results as the final results. Some system parameters in the experiments are referenced to EPC C1 Gen2 standard [16], and the others are referenced to the literature [11,12,15]. The detailed parameters are as follows.

- Channel: a flat fading linear time invariant channel during one identification cycle [12,15], the values of *h_n_*, *n* = 0,1, …*N* − 1 are random numbers from (0, 1] and *h_n_* ≠ *h_m_* when *n* ≠ *m*
- Nominal link frequency: *f_lp_* = 50 kHz [12,16]
- Symbol rate and delay: each tag’s symbol rate *a_n_* deviates up to ±22% from *f_lp_*, the symbol rate deviation among tags is also up to ±22%, and each tag’s symbol delay *b_n_* is less than 24 μs [11,16].
- Sampling frequency: 750 kHz
- Block length: The length *K* is 16 and identical to that of RN16 specified in EPC C1 Gen2 [16]
- Antenna: single receiving antenna
- The initial frame length: 128
- In LCE algorithm: When the number of collided tags is 2, 3, 4, and 5, *M* is 2, 3, 4, and 5.
- In OLCE algorithm: When the number of collided tags is 2, 3, 4, and 5, *J* is 120, 35, 15, and 5. The reason why *J* is chosen as such values is that, *J* needs to satisfy the orthogonal matrix condition and decrease with the number of collided tags

### 3.2. Estimation Error 

In order to evaluation the performance of channel estimation, we consider the relative error of channel estimation as the performance index under different signal to noise ratio (SNR), where the relative error is defined as:

(26) $$e=\left\|\hat{H}-H\right\|/\left\|H\right\|\times 100\%$$

in which ‖•‖ denotes the Euclidean norm, $\hat{H}$ is the estimated value of channel parameter and **H** is the set value of channel parameter. And SNR is defined as:

(27) $$\text{SNR}=E\left(\sum_{n=0}^{N-1}{\left|h_{n}c_{n}\right|}^{2}\right)/\sigma^{2}$$

**Figure 4:**
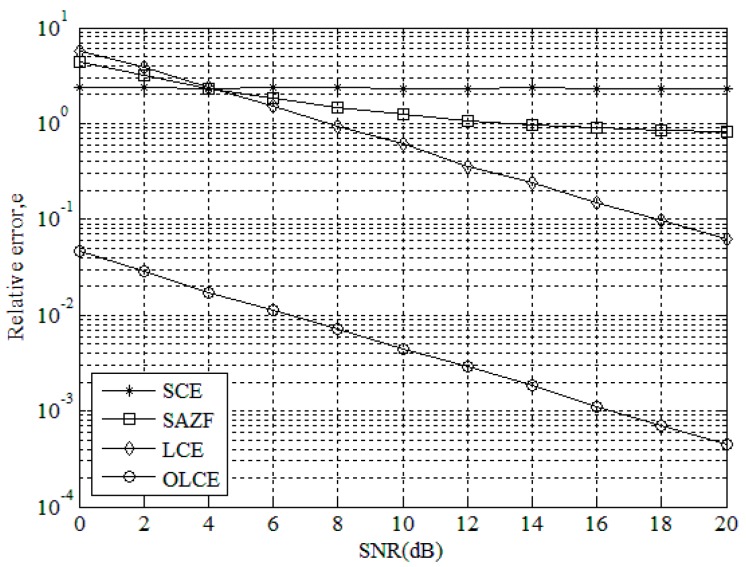
Relative error *e* of channel estimation for SCE, SAZE, LCE, and OLCE when the number of collided tags is three and SNR ranges from 0 to 20 dB.

Figure 4 gives the relative error *e* for SCE, SAZF, LCE, and OLCE when the number of collided tags is three and SNR ranges from 0 to 20 dB. In the figure, the three error curves of SCE, SAZF, and LCE are higher than 10° when SNR is smaller than 6 dB. This indicates that the performance of their channel estimation is poor under a small SNR range. The reason is that there are not only inter-tag interferences but also more noisy interferences. However, the error curve of OLCE is lower than 10^−1^. When SNR is 20 dB, the error curves of SAZF and SCE are higher than 10^−1^, LCE’s is lower than 10^−1^, and OLCE’s is lower than 10^−3^. What is more, when SNR is from 0 to 20 dB, the error curve of OLCE is always lower than the others. Since SAZF adopts the single receiving antenna, SAZF’s separation equation becomes indeterminate when the number of collided tags is beyond two. Hence, it does not work well. Since SCE adopts the SIC technique, cumulative errors will increase when the number of collided tags increases. Hence, the estimation errors will also increase. Since LCE adopts the inner product and its interferences will be accumulated. Therefore, SCE, SAZF, and LCE do not work better than OLCE under three collided tags.

**Figure 5:**
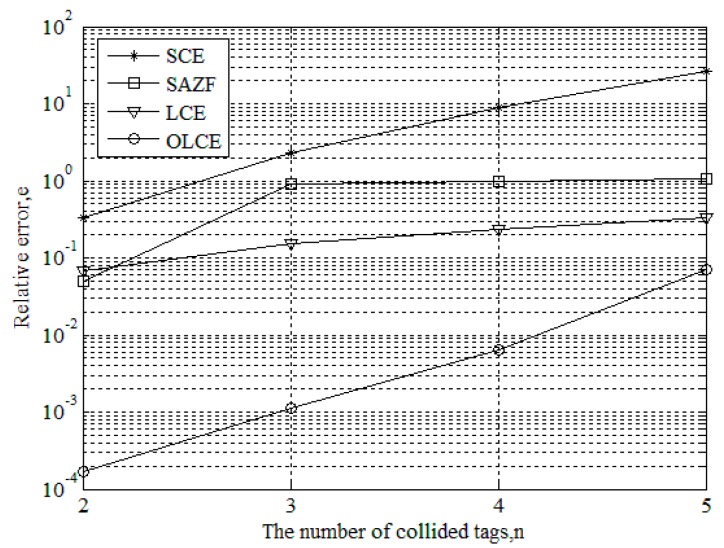
Relative error of channel estimation for SCE, SAZE, LCE, and OLCE when SNR is 16 dB and the number of collided tags ranges from two to five.

Figure 5 gives the relative error of channel estimation for SCE, SAZF, LCE, and OLCE when SNR is 16 dB and the number of collided tags ranges from two to five. From the figure, when the number of collided tags is two, the error curves of SCE, SAZF, and LCE are higher than 10^−2^, and OLCE’s is lower than 10^−3^. When the number of collided tags is beyond two, the error curve of OLCE is always lower than the others. Even though the number of collided tags is five, the error curve of OLCE is also below 10^−1^. This indicates that OLCE could guarantee the minimum MSE.

### 3.3. Separation Efficiency

In order to evaluate the performance of the signal separation, we consider separation efficiency under different signal to noise ratio (SNR), where the separation efficiency is defined as:

(28) $$P_{e}=n_{s}/n_{t}\times 100\%$$

where *n_s_* is the number of tags which are successfully separated, and *n_t_* is the total number of collided tags. In the experiment, a tag would be regarded as unsuccessful identification as long as there is one bit error.

**Figure 6:**
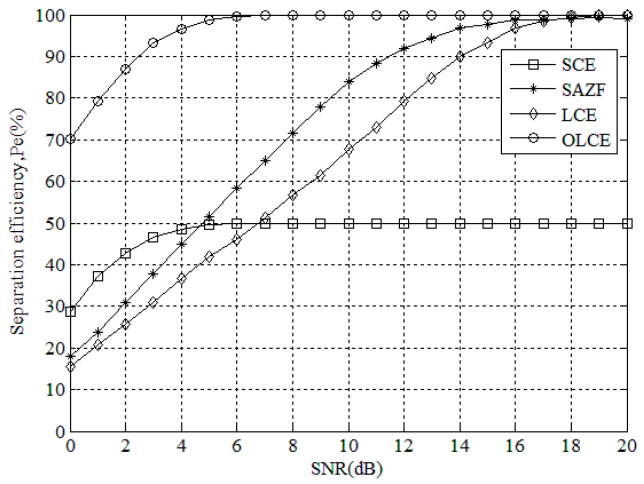
Separation efficiency for SCE, SAZE, LCE, and OLCE under different SNR when the number of collided tags is two.

Figure 6 gives the separation efficiency for SCE, SAZF, LCE, and OLCE under different SNR when the number of collided tags is two. From the figure, the separation efficiency of SCE is lower than SAZF and LCE’s when SNR is from 0 to 4 dB. The reason is that SCE separates collided tags by the strengths of tag signals. When the strengths of tag signals are very different, they are less affected by noise. When SNR increase, the separation efficiencies of SAZF and LCE are higher than SCE. The reason is that the accumulative errors make SCE separate only one tag whose signal strength is the highest. Thus, the maximum efficiency of SCE is only 50%. On the other hand, the separation efficiency of OLCE is higher than the others whatever SNR is. This means that OLCE has better estimation performance.

**Figure 7:**
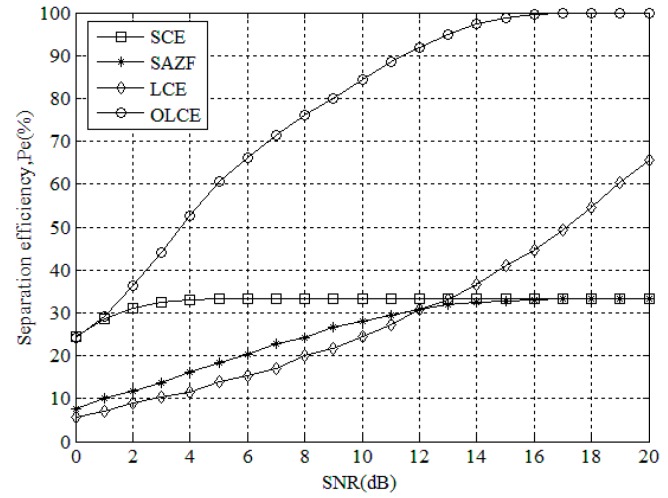
Separation efficiency for SCE, SAZE, LCE, and OLCE under different SNR when the number of collided tags is three.

Figure 7 gives the separation efficiency for SCE, SAZE, LCE, and OLCE under different SNR when the number of collided tags is three. From the figure, when SNR is from 0 to 13 dB, the separation efficiency of SCE is higher than SAZF and LCE. When SNR is from 14 to 20 dB, the separation efficiency of LCE is higher than SAZF and SCE. When SNR is 20 dB, the separation efficiencies of SAZF and SCE are about 35%, and LCE’s is about 65%. However, the separation efficiency of OLCE is always higher than the others, and its maximum efficiency achieve 100%. This result shows that OLCE works better than the others when the number of collided tag is three.

**Figure 8:**
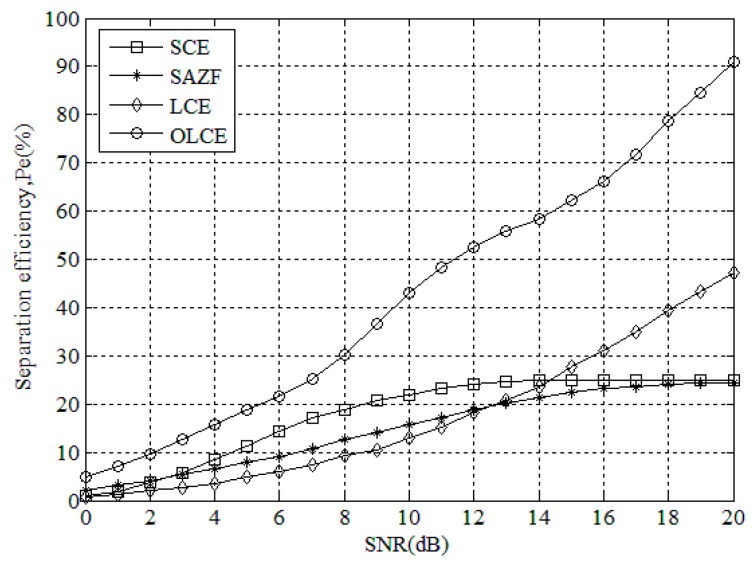
Separation efficiency for SCE, SAZE, LCE, and OLCE under different SNR when the number of collided tags is four.

Figure 8 gives separation efficiency for SCE, SAZE, LCE, and OLCE under different SNR when the number of collided tags is four. From the figure, the separation efficiency of OLCE is always higher than SCE, SAZF, and LCE. The maximum efficiency of SCE and SAZF could achieve is only 25%, which means the two algorithms successfully separate only one tag at most. LCE’s maximum efficiency is close to 50%. On the contrary, OLCE’s maximum efficiency is close to 100%. This means that it could nearly separate all of collided tags. From this result, OLCE also works better than SCE, SAZF, and LCE when the number of collided tags is four.

### 3.4. STR Performance

The performance of tag identification is greatly improved by using the separation technique. In the cross-layer approach, the collided time slot is no longer considered as useless. Thus, it has higher identification efficiency than the pure MAC layer method. In this paper, we consider the number of successful identification tags to the number of total time slots ratio (STR) as the measurement of recognition performance, where STR is defined as:

(29) $$\text{STR}=N_{s}/N_{L}$$

in which *N_s_* is the number of successful identification tags, and *N_L_* is the number of total time slots. For the cross-layer approach in this numerical experiment, the MAC algorithm is chosen as dynamic frame slot Aloha (DFSA), and the channel estimation on the PHY layer is SCE, SAZF, LCE, and OLCE, respectively. 

**Figure 9:**
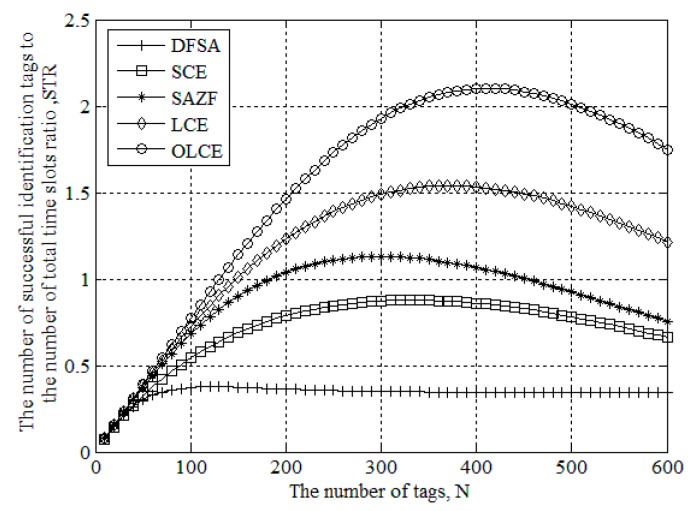
STR for DFSA, SCE, SAZF, LCE, and OLCE under different the number of tags when SNR is 20 dB.

Figure 9 gives STR for DFSA, SCE, SAZF, LCE, and OLCE under the different number of tags when SNR is 20 dB. In Figure 9, we can see that the STR of the cross-layer approach (SCE, SAZF, LCE, and OLCE) is higher than the STR of the pure MAC layer approach (DFSA). Except that the maximum of DFSA’s STR is less than 0.4, the maximum of the others is greater than 0.85. The reason is that the successful identified tags in the cross-layer approach may be both on the PHY layer and the MAC layer. For the pure MAC_layer approach, only a tag is identified in a slot, so STR is not more than 1. For the cross-layer approach, two or more tags can be separated in a slot, so STR can be greater than 1. Furthermore, we can see that the STR cure of OLCE is higher than the other curves.

## 4. Conclusions

In this paper, we propose a novel algorithm for the recovery of UHF RFID tag collision. The algorithm uses MMSE criterion and has lower estimation errors than the existing algorithms. Adopting the algorithm, we have higher separation efficiency on the PHY layer. Moreover, the algorithm still has superior separation efficiency even when the number of collided tags is beyond two. In addition, we show that the STR performance of the cross-layer approach using the proposed algorithm would outperform the existing cross-layer approach. The proposed algorithm’s maximum STR is more than 2.1.
